# Diagnosis and Management of Acute Coronary Syndrome Patients Without Obstructive Epicardial Stenosis

**DOI:** 10.1007/s11886-025-02191-1

**Published:** 2025-01-30

**Authors:** Khaled Ziada, Hassan Alkhawam, Haidar Hajeh, Malak Modi, Tarek Helmy

**Affiliations:** 1 Department of Cardiovascular Medicine, Cleveland Clinic, 9500 Euclid Avenue, Cleveland, Ohio 44195 USA; 2 Department of Cardiovascular Disease, University of Alabama, 2700 10th Ave S #305, Birmingham, AL 35205 USA; 3 Medicine Department, UCLA Kern Medical, Bakersfield, CA 93305 USA; 4 Division of Cardiology, Louisiana State University Health Sciences Center - Shreveport (LSUHSC-S), 1501 Kings Hwy, Shreveport, LA 71103 USA

**Keywords:** MINOCA, INOCA, Coronary microvascular dysfunction, SCAD, Coronary vasospasm, Coronary functional assessment

## Abstract

**Purpose of Review:**

What is the pathophysiology and clinical findings as well as management of patients presenting with INOCA/MINOCA (Ischemia/Myocardial Infarction with Non-Obstructive Coronary Arteries).

**Recent Findings:**

INOCA/MINOCA has a complex pathophysiology.

**Summary:**

In this review article, we aim to summarize the complex pathophysiology and clinical diagnosis, and review the current management options.

## Introduction

Myocardial infarction (MI) is a critical and life-threatening condition that occurs when there is an inadequate blood supply to the heart muscle, leading to injury and death of myocardial cells. The most common cause of MI is obstructive coronary artery disease (CAD), a condition characterized by the significant narrowing (> 50%) or complete occlusion of one or more coronary arteries due to the progressive buildup of atherosclerotic plaques and plaque rupture. However, a subset of patients present with myocardial infarction in the absence of significant coronary artery obstruction (i.e. stenosis of < 50%), a condition known as Myocardial Infarction with Non Obstructive Coronary Artery Disease (MINOCA). MINOCA represents a diagnostic challenge and necessitates a unique understanding of its pathophysiology, clinical manifestations, diagnostic modalities and management strategies.

MINOCA is being increasingly recognized as an important clinical entity, accounting for a significant proportion of acute myocardial infarctions. The prevalence of MINOCA varies widely depending on the studied population, ranging from approximately 5–15% of all patients with MI. This condition predominantly affects women and tends to occur in younger individuals compared to those with Acute myocardial infarction with obstructive CAD (AMI-CAD) [1–3].

MINOCA is diagnosed when the patient presents with clinical features suggestive of myocardial infarction such as chest pain, electrocardiographic changes indicative of ischemia, and elevated cardiac biomarkers, in the absence of significant epicardial coronary artery stenosis (less than 50% luminal narrowing). It is necessary to rule out other causes of elevated cardiac biomarkers including demand ischemia, sepsis, pulmonary embolism, anemia etc. before establishing the diagnosis of MINOCA [1].

Although initially considered a benign condition, growing evidence suggests that MINOCA is associated with adverse outcomes including recurrent cardiovascular events with a frequency that is comparable to CAD-associated MI [1] in addition to high in-hospital and long-term mortality rates [4].

The underlying pathophysiology of MINOCA is complex and multifactorial. Several mechanisms contribute to the development of myocardial infarction in the absence of significant coronary artery obstruction. Understanding these mechanisms is crucial for accurate diagnosis, risk stratification, and targeted interventions. In recent years, research efforts have shed light on various pathophysiological processes involved in MINOCA, including coronary microvascular dysfunction, plaque disruption and embolization, coronary artery spasm, non-atherosclerotic causes, and other factors.

This article aims to provide a comprehensive overview of the pathophysiology of MINOCA, elucidating the intricate interplay of contributing mechanisms and reviewing the current data and management options.

## Epidemiology

The epidemiology of MINOCA reveals important insights into the prevalence and clinical characteristics of this condition. MINOCA accounts for a significant proportion of acute MI. Multiple studies have investigated the prevalence of MINOCA which varies between 5% and 15% of all patients with MI [2–4]. Patients diagnosed with MINOCA are more likely to be females presenting at a younger age as compared to patients with AMI-CAD. Among females, MINOCA patients were more likely to have hypercoagulable state but lacked traditional cardiac risk factors including hypertension, diabetes, etc [2].

In addition to age and sex, some studies have shown that MINOCA is more commonly observed in the nonwhite population compared to AMI-CAD [2, 3].

Different studies comparing the prevalence of common MI risk factors between MINOCA and patients with AMI-CAD including hypertension, diabetes, dyslipidemia, and smoking have shown heterogeneous results. Large studies however showed a statistically significant lower prevalence of these risk factors in the MINOCA group [2, 3].

MINOCA poses a significant burden on healthcare systems and is associated with adverse outcomes and significant morbidity and mortality. In-hospital and 12 months mortality rates in MINOCA patients however have been reported to be lower than what is observed in MI patients with obstructive CAD [3, 4]. No sex difference was observed in in-hospital mortality rates in MINOCA patients [3].

Even though, prognosis of patients with MINOCA is better compared to AMI-CAD patients, the 12-month all-cause mortality in MINOCA patients is 4.7% [4]. Poor prognosis associated with MINOCA highlights the importance of accurate diagnosis, appropriate risk stratification, and targeted management strategies to improve patient outcomes. Further research is required to better understand the prognostic factors and optimize the management of MINOCA to reduce the risk of future events.

## Pathophysiology

The pathophysiology of MINOCA involves multiple pathological processes that work independently or in tandem. In addition to atherosclerotic causes like plaque disruption, non-atherosclerotic causes like coronary microvascular dysfunction, coronary artery spasm, sudden coronary artery dissection and coronary artery embolism have been identified to play a role in pathophysiology of MINOCA. Many of these processes are not detectable using conventional coronary angiography leading to the absence of obstructive coronary artery disease.

## Plaque Disruption

Coronary arterial flow compromise in MINOCA can happen as a result of atherosclerotic plaque compromise [5]. These events might be difficult to detect on coronary angiography which may show normal coronaries or non-obstructive disease, but more sensitive techniques using intravascular ultrasound (IVUS) [6] or optical coherence tomography (OCT) may detect these abnormalities. Plaque rupture was encountered in 37% of MINOCA patients in a study evaluating acute coronary syndrome patients with IVUS indicating a significant role of plaque dysfunction in the MINOCA population [7]. These plaques may exhibit features such as a large lipid core and a thin fibrous cap leading to increased vulnerability to rupture [8].

## Coronary Microvascular Dysfunction (CMD)

CMD encompasses structural and functional abnormalities at the level of small-caliber coronary arteries that compromise coronary blood flow leading to myocardial ischemia. Studies have shown that CMD plays an important role in the pathophysiology of MINOCA [9]. Proposed mechanisms of CMD include a myriad of functional and structural abnormalities that involve oxidative stress, hormonal imbalances, endothelial dysfunction, and microvascular spasm [10]. This leads to the activation of inflammatory pathways and increased production of pro-inflammatory cytokines and oxidative damage, contributing further to endothelial damage and microvascular dysfunction in a vicious cycle fashion [11]. CMD can be evaluated by non-invasive modalities (like positron emission tomography (PET) or cardiac magentic resonance imaging (MRI)) in addition to invasive tests.

## Coronary Artery Spasm

Transient and reversible coronary artery spasm can contribute to the pathophysiology of MINOCA. Spasm of the epicardial coronary arteries causes abrupt reduction or cessation of blood flow, leading to myocardial ischemia and subsequent infarction. Some studies have reported the presence of coronary artery spasm in up to 50% of MINOCA patients [12]. The exact mechanisms triggering coronary artery spasm in MINOCA are not fully elucidated but may involve response to vasospastic agents including cocaine, methamphetamine, cigarette smoking, or other unknown intrinsic factors. This leads to dysregulation of vascular smooth muscle tone, enhanced sympathetic activity, impaired nitric oxide-mediated vasodilation and altered endothelin release. The resulting imbalance between vasodilatory and vasoconstrictive processes leads vascular smooth muscle hyperreactivity causing coronary vasospasm and transient myocardial ischemia [13]. Diagnosis of coronary artery spasm usually involves provocative testing using acetylcholine or ergonovine to induce spasm [14].

## Spontaneous Coronary Artery Dissection (SCAD)

SCAD is defined as a spontaneous tear in the coronary artery wall with potential mural hematoma and possible luminal compromise. Though, the underlying mechanism of SCAD is not exactly known, it is believed to be precipitated by stressor mediated catecholamine surge in the setting of underlying vasculopathy. Some cases of SCAD can be detected on coronary angiography, but caution should be exercised to avoid extending the dissection with repeated injections or intervention [15].

## Coronary Artery Thrombosis or Embolism

Thrombosis or embolism of coronary artery can happen in patients with hypercoagulable state. When it involves coronary microcirculation, without evidence of obstructive disease on coronary angiography, it can present as MINOCA. Hypercoagulable states including inherited thrombophilia, thrombotic thrombocytopenic purpura (TTP), antiphospholipid antibody syndrome, heparin induced thrombocytopenia, and myeloproliferative diseases have been associated with MINOCA [1].

Other contributing factors that play a role in MINOCA development include vasculitis, myocardial inflammation or stunning, coronary microthrombi and microvascular endothelial dysfunction. Myocardial inflammation is driven by various etiologies such as autoimmune disorders or viral infections. Myocardial stunning is reversible myocardial dysfunction following an ischemic insult. Coronary microthrombi can impede blood flow in the microcirculation, exacerbating myocardial ischemia. Microvascular endothelial dysfunction, characterized by impaired vasodilation and increased endothelial permeability, may further compromise coronary blood flow.

## Diagnosis

**Fig. 1:**
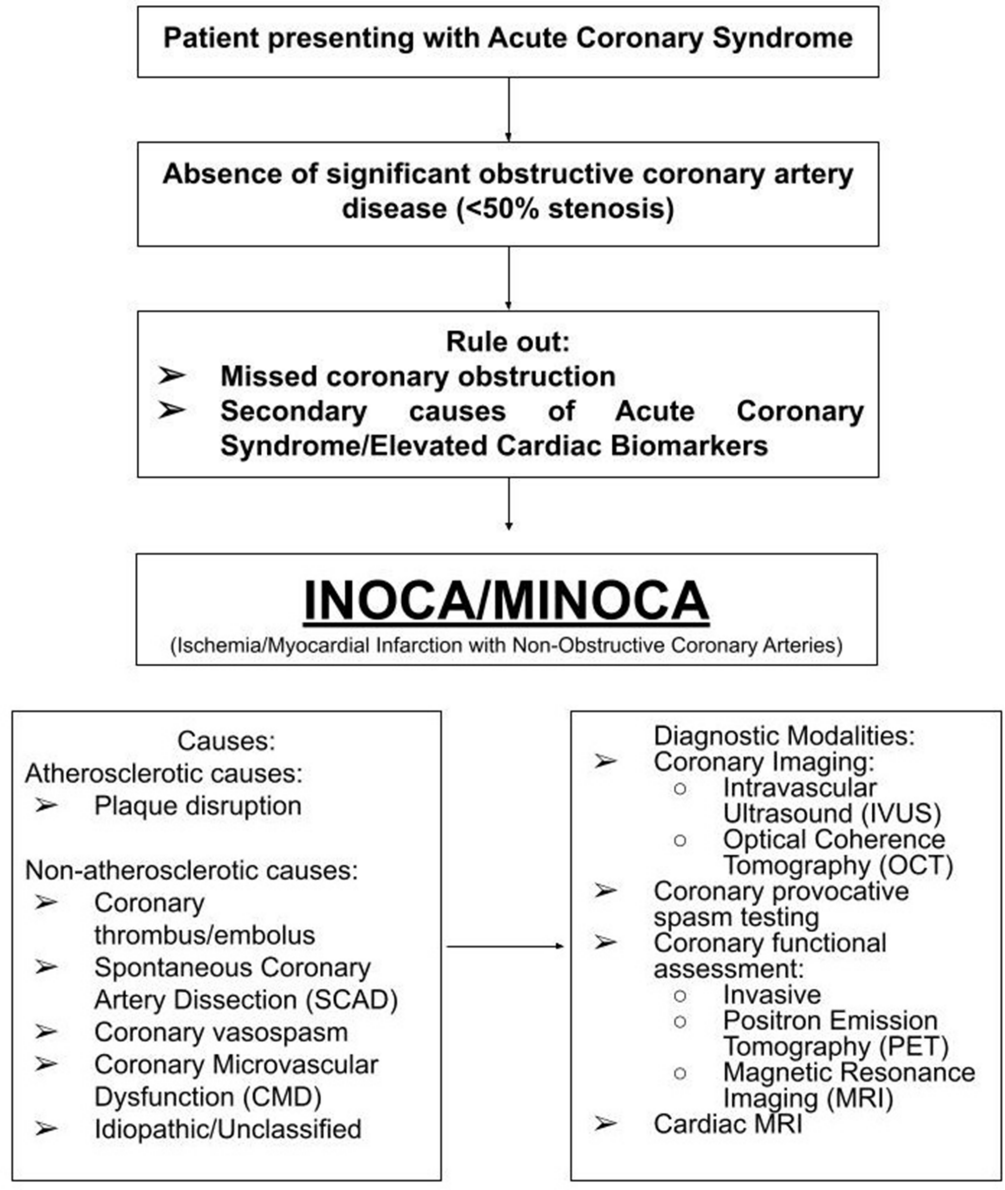
Clinical presentation, causes, and diagnosis of MINOCA (Myocardial Infarction with Non-obstructive Coronary Arteries)

The diagnosis of MINOCA is established in cases of acute coronary syndrome (Chest pain or equivalent symptoms, ischemic EKG changes in the form of ST elevation or more commonly ST depression [4] and/or elevated cardiac biomarker levels) and coronary angiography showing a non-obstructive coronary pattern with the absence of a ‘culprit’ lesion to explain the clinical presentation. It is imperative to note that, MINOCA is a diagnosis of exclusion, hence secondary causes of elevated cardiac biomarkers should be ruled out as well (Fig. 1).

A basic and important diagnostic step is to investigate the presence or absence of atherosclerosis (intimal wall thickening) and luminal narrowing that may have been missed on routine angiography. Even though IVUS can be used to study atherosclerotic plaque, it is limited in its ability to detect plaque erosion. OCT is extremely helpful in this setting. It is the modality of choice given its high resolution, particularly in the superficial layers of the coronary wall where the underlying pathology is frequently located [16]. OCT can detect signs of atherosclerosis raising the possibility of plaque erosion, plaque rupture or microvascular dysfunction as the main etiology [17]. The absence of atherosclerosis, however, may suggest other pathophysiologies mentioned above as an explanation of the MINOCA presentation [18].

Plaque erosion is typically seen as a non-disrupted plaque, more likely with a lipid core, with an intact fibrous cap and an overlying luminal thrombus. The thrombus is usually small and the infarct size is not large, though there are exceptions. Plaque erosion can occur with mild non-obstructive plaques, which is the likely scenario in MINOCA cases. About one third of MINOCA cases can be explained by plaque erosion [19]. Plaque rupture on the other hand is diagnosed when there is a disruption in the continuity of the fibrous cap with evidence of intraluminal thrombus. Commonly the thrombus is larger and ‘craters’ within the body of the plaque representing the site of the lipid core before rupture. OCT has a higher resolution compared to IVUS and is the modality of choice when available. In 20–25% of cases with an apparent atherosclerotic lesion and luminal thrombus, it is not possible to distinguish between erosion and rupture on OCT imaging due to the subtlety of the findings and/or presence of a red thrombus, which does not allow the penetration of the light beam, thus not allowing visualization of the underlying plaque [20].

In the absence of atherosclerosis, imaging can elucidate intraluminal thrombus which may have embolized from an extra-coronary source, or in rare cases of spontaneous in-situ thrombosis due to hypercoagulability.

Non-atherosclerotic arteries in MINOCA patients can exhibit signs of SCAD, which has been reported in about 5% of MINOCA patients [21]. SCAD is usually diagnosed with coronary angiography. Entry points into the false channel of the dissection or flap can sometimes be identified on OCT [22]. Importantly, advancing guidewires and imaging catheters in cases of SCAD can be consequential if the wire or imaging catheter go from the true to the false lumen, extending the false channel distally.

Angiographic findings of SCAD can be divided into three types. Type 1 SCAD represents the classic arterial contrast staining with a visible false lumen. In type 2 or 3 SCAD however, luminal abnormalities may be subtle or absent with no visible dissection flap. Type 2 SCAD refers to the presence of long diffuse intramural hematoma leading to a smooth narrowing of a coronary artery segment. Type 3 on the other hand refers to multi-focal intramural hematomas [23]. SCAD type 2 and 3 may require further imaging with optical coherence tomography (OCT) as they may be missed on angiography [22].

Most of the imaging findings in MINOCA represent transient phenomena and may not be visualized if imaging is not performed acutely. In case of plaque erosion for example, the overlying thrombus may embolize or be absorbed. Similarly, the intramural hematoma of SCAD also gets absorbed and the flap at the entry point heals. Recent evidence suggests that the yield of imaging is highest if performed within 2 weeks of the acute event, with findings becoming less detectable with time [24].

Coronary vasospasm is another common cause of MINOCA. Provocative testing during invasive angiography remains the most reliable method to make the diagnosis of coronary spasm, as there are no available standardized non-invasive tests to make the diagnosis. Provocation can be performed by intracoronary infusion or slow IV injection of small escalating doses of acetylcholine or by IV administration of ergonovine [25]. Angiograms, electrocardiographic changes and patient symptoms are monitored to assess for evidence of spasm. Diagnosis of coronary vasospasm is made when there is a significant narrowing of the coronary artery with provocation testing along with accompanying signs of ischemia (EKG changes) [26].

Provocation in the acute setting may not always reflect accurate susceptibility to spasm, given heightened inflammatory state and circulation of vasoactive cytokines. Additionally, initiation of vasodilator therapies (such as nitrates and/or calcium channel blockers) prior to arrival to the catheterization laboratory reduces the sensitivity of the test.

In cases of MINOCA caused by coronary microvascular dysfunction (CMD), intracoronary imaging will not reveal an underlying mechanism for the acute presentation. In such cases, invasive testing can be performed in the catheterization laboratory immediately after coronary angiography. The basis for the diagnosis is the demonstration of an abnormal coronary flow reserve (CFR) and/or elevated microcirculatory resistance. Of note, it is important to document normal epicardial coronaries before pursuing these invasive diagnostic methods as they do not differentiate between abnormal flow from epicardial disease versus CMD. CFR < 2.0 with the use of vasodilatory agents like adenosine suggests impaired coronary flow [26]. Coronary slow flow phenomenon may also be seen in patients with increased basal coronary microvascular resistance suggestive of CMD.

CFR is calculated based on the ratio of maximum hyperemic to basal coronary flow. Traditionally, CFR assessment has been performed using either Doppler flow velocity or a bolus thermodilution method. The initial approach to measure Doppler CFR was to use a doppler tipped wire advanced to the mid to distal vessel to calculate the average peak velocity of coronary flow, first at rest, then after induction of hyperemia using adenosine. The calculated velocity ratio is an approximated flow ratio. If the distal pressure at peak hyperemia is known (using a solid state pressure sensor), the hyperemic microcirculatory resistance (hMR) can be calculated as well. The bolus thermodilution technique method is another method utilized to calculate CFR. In such cases, the dedicated wire has a solid-state pressure sensor and a thermistor near its tip. Injecting a bolus of cold saline in the proximal coronary artery and plotting the temperature change curve sensed by the distal thermistor defines a transit time of flow between the 2 points in the artery. Repeating the calculation of the transit time at peak hyperemia induced by adenosine (typically administered via an IV infusion) then defines the transit time of flow at peak hyperemia. The ratio between the 2 transit times expresses that of CFR [27].

$$\:\mathrm{C}\mathrm{F}\mathrm{R}\hspace{0.17em}=\hspace{0.17em}{\mathrm{Q}}_{\mathrm{h}\mathrm{y}\mathrm{p}\mathrm{e}\mathrm{r}}/{\mathrm{Q}}_{\mathrm{r}\mathrm{e}\mathrm{s}\mathrm{t}}\:=\:{\mathrm{T}}_{\mathrm{r}\mathrm{e}\mathrm{s}\mathrm{t}}/{\mathrm{T}}_{\mathrm{h}\mathrm{y}\mathrm{p}\mathrm{e}\mathrm{r}}$$

Where Q_hyper_ and Q_rest_ is the coronary flow at hyperemia and rest respectively and T_hyper_ and T_rest_ represents mean transit time of the indicator (cold saline) at hyperemia and rest.

As the distal wire is also equipped with a pressure sensor, the distal pressure and transient time at peak hyperemia can be integrated to calculate the index of microcirculatory resistance (IMR) [27].

$$\:\mathrm{I}\mathrm{M}\mathrm{R}\:=\:\mathrm{P}_\mathrm{d} \times \mathrm{T}$$

(where P_d_ is the distal coronary pressure and T is the mean transit time at peak hyperemia)

It is difficult to define a normal CFR value due to the wide range and its dependence on loading conditions. Historically however, a CFR ≤ 2.0 was considered abnormal [26, 28]. Comparative studies suggest that the equivalent cutoff using thermodilution is closer to 2.5 [29]. hMR > 1.9 or 2.0 is considered abnormal and IMR > 25 is abnormal in the elective setting.

Even though both Doppler CFR and bolus thermodilution methods have inter as well as intra-observer variability, these methods are widely used. To overcome the observer variability and overcome the operator bias, a novel technique with continuous thermodilution has been recently validated [27, 30–32].

The continuous thermodilution method is based on the assumption that the indicator (cold saline) is homogenously mixed with the blood. It determines the absolute flow (Q) as follows:

$$\:\mathrm{Q}\hspace{0.17em}=\hspace{0.17em}1.08\:[{\mathrm{T}}_{\mathrm{b}}-{\mathrm{T}}_{\mathrm{i}}\:/\:{\mathrm{T}}_{\mathrm{b}}-\mathrm{T}]\:{\mathrm{Q}}_{\mathrm{i}}$$

Where Q_i_ is the continuous infusion rate of saline at room temperature, T is the temperature measured in the distal segment of coronary (temperature of blood mixed with cold saline), T_b_ and T_i_ is the temperature of blood and saline respectively while entering the coronary circulation and 1.08 is the constant representing the density and specific heart of blood and saline.

Further absolute microvascular resistance can be calculated as

$$\:\mathrm{R}\hspace{0.17em}=\hspace{0.17em}{\mathrm{P}}_{\mathrm{d}}\:/\:\mathrm{Q}$$

Though relatively new, this approach with continuous thermodilution presents an opportunity to better understand the coronary microvasculature and can be useful to evaluate for CMD.

In addition to invasive assessment, non-invasive measurements using PET or MRI stress and rest scans can also aid in detection of coronary microvascular dysfunction [33]. The PET myocardial blood flow ratio (MBFR) follows a very similar principle to what is described above [33]. Myocardial perfusion reserve index (MPRI) is a similar measurement obtained by cardiac magnetic resonance imaging, though not widely utilized due to the need for dedicated quantitative assessment of perfusion.

Non-invasive cardiac imaging including but not limited to cardiac MRI can be useful in distinguishing MINOCA from other ischemic or non-ischemic conditions that can mimic MINOCA such as myocarditis, Takotsubo syndrome or acute presentation of cardiomyopathy. Myocarditis and stress cardiomyopathy are not rare and should be high on the list of differential diagnoses. MRI imaging can reveal myocarditis in up to one third of the MINOCA presentations [34].

Management.

Due to the scarcity of literature supporting a standardized medical regimen for MINOCA management, clinical practice still relies on observational studies and small randomized trials. In an observational study on 9466 MINOCA patients from the SWEDEHEART registry with a primary endpoint of major adverse cardiac events, results showed lower events in the statin group and the angiotensin-converting enzyme inhibitors (ACEi)/ angiotensin receptor blockers (ARB) group. Beta-blockers showed a hazard ratio of major adverse events of 0.86 (0.74–1.01) suggesting a positive trend. Dual antiplatelet therapy in this study however did not show a lower event rate [35]. The ongoing MINOCA-BAT trial is expected to shed more light on the role of beta-blockers and ACEis/ ARBs in the management of MINOCA patients [36]. It is to be noted that, in all MINOCA patients, aggressive risk factor modification is recommended.

Since, by definition, MINOCA indicates no evidence of obstructive disease, the need for revascularization has been questioned. Even in cases where plaque rupture or plaque is thought to be the etiology of MINOCA, it is not a routine practice to perform percutaneous coronary intervention. However, antiplatelet therapy with aspirin and statin are strongly recommended in these patients. But the role of dual antiplatelet therapy is debatable, especially from the results of SWEDEHEART study [35].

Similarly, in SCAD, intramural hematomas generally heal over weeks and intervention can cause dissection extension with well documented low rates of success [37]. Beta blockers and control of hypertension are the most critical therapeutic approaches [38]. However, in acute lumen compromise, the use of a cutting balloon to “fenestrate” the hematoma may be beneficial.

In patients with coronary thrombosis or embolism, the mainstay of treatment is antithrombotic and antiplatelet therapies. However, more research is needed to guide optimal duration of antiplatelet therapy in these patients. It should be noted that secondary causes of coronary thrombosis including hypercoagulable states should receive specific adjunctive therapies.

CMD is typically treated with beta blockers or calcium channel blockers as first line therapy for symptomatic relief. Ranolazine serves as a second line treatment with anecdotal but no well-documented success in improving outcomes. Controlling risk factors such as statins for dyslipidemia, smoking cessation and control of hypertension and diabetes are critical in restoring endothelial function integrity. Non-pharmacologic approaches such as cardiac rehabilitation, stress reduction and weight loss have demonstrated varying degrees of success in improving symptoms and likely longer-term outcomes [26]. Since data for treatment of CMD in MINOCA patients is limited, large clinical trials are required to guide further management.

Coronary spasm is treated with calcium channel blockers and nitrates, separately or in combination. Similar to CMD, these patients frequently have evidence of atherosclerosis and control of risk factors is essential in reducing future risk of disease progression [26]. In cases of CMD and/or coronary spasm, there is frequent overlap of pathophysiologic mechanisms. In the CorMICA trial, up to 20% of patients with ischemia with nonobstructive coronary artery disease had evidence of both CMD and spasm [39]. The study demonstrated that using invasive testing results in the diagnosis of the underlying pathology and tailoring the treatment accordingly, which resulted in improvement in quality of life and better symptom control [39]. Importantly, this study was geared towards patients with stable syndromes, not ACS.

## Conclusions

In summary, there are no randomized trials for management of MINOCA patients. Hence, their treatment should be focused on underlying etiology of the disease. In general, most patients will benefit from antiplatelet agents, statins, ACEI/ARB and betablockers depending on underlying mechanism of MINOCA in each patient. Patients with evidence of coronary vasospasm should be treated with calcium channel blockers and nitrates. All patients with underlying atherosclerosis should be treated aggressively for modifiable risk factors.

## Key References

- Tamis-Holland JE, Jneid H, Reynolds HR, Agewall S, Brilakis ES, Brown TM, et al. Contemporary Diagnosis and Management of Patients With Myocardial Infarction in the Absence of Obstructive Coronary Artery Disease: A Scientific Statement From the American Heart Association. Circulation [Internet]. 2019 [cited 2023 Dec 20];139:E891–908. Available from: https://www.ahajournals.org/doi/abs/10.1161/CIR.0000000000000670.

This is the Scientific Statement from the American Heart Association.

- Candreva A, Gallinoro E, van ‘t Veer M, et al. Basics of Coronary Thermodilution. JACC Cardiovasc Interv. 2021;14(6):595–605. 10.1016/j.jcin.2020.12.037.

This article explains the basics of thermodilution and coronary functional assessment.

## Data Availability

No datasets were generated or analysed during the current study.
